# Effect of health education on knowledge and attitude of menopause among middle-age teachers

**DOI:** 10.1186/s12905-020-01095-2

**Published:** 2020-10-12

**Authors:** Helen Gebretatyos, Lidia Ghirmai, Soliana Amanuel, Ghidey Gebreyohannes, Zemenfes Tsighe, Eyasu H. Tesfamariam

**Affiliations:** 1 Department of Midwifery, School of Nursing, Asmara College of Health Sciences, Asmara, Eritrea; 2 Dean of Asmara College of Health Sciences, Asmara, Eritrea; 3 Higher Education administration and International Linkages, NHERI, Asmara, Eritrea; 4 Department of Statistics, Biostatistics and Epidemiology Unit, College of Science, Mai Nefhi, Eritrea

**Keywords:** Menopause, Knowledge, Attitude, Health education

## Abstract

**Background:**

Adequate knowledge and positive attitude toward menopause are important for women to tackle changes related to menopause. Even though all women experience menopause at some stage in their life, teachers face more difficulties more than other female employees due to the nature of their roles do. In Eritrea, menopause has been given little attention hence gaps exist concerning women’s knowledge, attitude, and the effects of health education on the same subject. This study aimed at assessing the effect of health education on knowledge and attitude of menopause among middle-aged teachers in elementary, junior, and secondary schools of Asmara, Eritrea.

**Method:**

A semi-experimental design with pre-intervention, immediate post-intervention, and three-month follow up test was used in this study. The data was collected from 99 middle age teachers using stratified random sampling. The intervention was done using lectures, group discussions, brochures, and handouts. Data on socio-demographics, knowledge, and attitude was collected using a pre-designed questionnaire. The effect of educational training at the three-time points was evaluated by repeated measure ANOVA using SPSS version 22.

**Results:**

The mean scores of correct knowledge at pre-intervention, immediate post-intervention, and 3-months follow-up were 12.3/22 (SD = 3.06), 17.3/22 (SD = 3.21), and 16.5/22 (SD = 2.52) respectively. A significant difference in scores of knowledge at the three-time points was observed due to the educational intervention with a statistical significance of (*p* <  0.0001). Post-hoc analysis revealed that knowledge score immediately after intervention was significantly greater than that of pre-intervention (*p* <  0.0001), and 3-months follow-up (*p* = 0.004). The mean scores of attitude at the three-time points were 27.9/45 (SD = 5.14), 28.3/45(SD = 5.25), 28.32/45(SD = 5.12). The educational intervention had brought a change in the mean scores of attitude at the three-time point with a statistical significance of (*p* < 0.0001). Post-hoc analysis revealed that attitude scores at immediate post-intervention were also significant (*p* = 0.001) with the 3-months follow up at (*p* < 0.0001) were higher than that of pre-intervention.

**Conclusion:**

The structured educational intervention was beneficial to the studied women in intensifying their knowledge and tuning them toward a positive attitude. Hence, proper health education programs regarding menopause are strongly recommended.

## Background

Middle age is the period in which most women face various changes. These changes could be social, physiological, psychological, and economical. On the other hand, several chronic diseases such as hypertension, diabetes, arthritis, and heart diseases have a high tendency of occurrence during the middle age [1]. Besides, it is the period in which women face menopause and it’s symptoms [2]. Even though natural, menopause might affect women’s health and their quality of life [3–6].

Menopause is the permanent cessation of menstruation that is recognized after 12 months of amenorrhea. It occurs due to a decline in the production of ovarian gonadotropins, estrogen, and progesterone [7]. This brings vasomotor and psychological symptoms [8]. Some women could also face health problems related to menopause such as cardiovascular diseases and osteoporosis [9]. Many factors play a role in bringing about those complications. One of these factors is age as a natural phenomenon driven by genetic and environmental causes. Generally, the median age at natural menopause is 51 years and varies in a different population [2, 10].

An upward trend in life expectancy has led women to spend a third of their lives in post- menopause [11], in addition the improvement in standards of living has been affecting the rate of perimenopausal and postmenopausal women [12]. This leads to an increased involvement of middle-age women in the workforce [13]. Various studies have revealed that menopausal symptoms predominantly affect women in the workforce than those out of the workforce [14–17]. This might be due to an increase in work stress and working environment [15]. Teaching is a profession that demands high concentration, memory, and stable mood which are highly affected by menopause [18]. Therefore middle age teachers require timely attention [19].

Knowledge about the definition of menopause, age at menopause, and various symptoms of menopause is helpful to adapt to this stage of life, as it is an unavoidable life event [20–25]. Consequently, women’s favorable attitude toward menopause also has a great effect on reducing the effects of menopausal symptoms [26]. Several studies have shown women have inadequate knowledge and negative attitude towards menopause [19, 20, 22–24, 27–33]. According to these studies, the lack of awareness and limited access to proper knowledge of menopause and its symptoms is being augmented by the delivery of contradictory information and social rumors [20, 22, 23].

Health education is one of the many ways that can be employed on the improvement of women’s knowledge and attitude towards menopause. Moreover, this can empower women to control their own lives [34]. Studies done in Egypt have also revealed that women’s knowledge and attitude has significantly increased after the health educational intervention [20, 23].

So far, in Eritrea, women’s health promotion policies and programs are specifically limited to issues of pregnancy and family planning. Moreover, Eritrean culture does not motivate women to discuss issues of menstruation, sexuality, or menopause openly even with their friends and family. Furthermore, Studies related to menopause in Eritrean women have not been published yet. As menopausal experience and the attitude towards menopause vary with the culture it is logical to think, the effect of menopause in Eritrean women might be unique to this group of women [35]. Therefore, this study aimed at investigating the effect of health education on knowledge and attitude of middle age teachers toward menopause. According to the results of this study, the methods used could be replicated in improving Eritrean women’s quality of life during the menopausal transition and beyond.

## Method

### Design, study setting, and population

A semi-experimental design with pre-intervention, immediate post-intervention, and three-month follow-up test was used in this study, conducted from June to October 2018 in Asmara, Eritrea. Asmara is the capital city and largest settlement in Eritrea, home to a population of 416,367 people according to records in the municipality of Asmara. At the time of the study, there were 677 female teachers aged 40 to 60 years in elementary (*n* = 562), junior (*n* = 87), and secondary (*n* = 28) schools.

### Sampling

The sample size was calculated using a G-power (Version 3) calculator based on priori by giving α, power, and effect size. Matched pairs were selected as the test family in the test software. The input required was tails (two), effect size (0.3), actual power (0.80), and level of significance (0.05) which resulted in a sample size of 90 women. Considering a 10% dropout from the follow-up, finally, 99 females were found to be adequate. Homogeneous knowledge and attitude of the female teachers within each of the levels of education, namely, elementary, middle, and secondary schools was observed. However, there were differences in knowledge and attitude among the three levels of education. Hence, stratified random sampling was used to select female teachers using proportional allocation. The selected size of the sample of female teachers from elementary, middle, and secondary levels was 82, 13, and 4 respectively.

### Intervention

Step 1: Permission was attained from the research ethical committee of Asmara College of health sciences and the zonal office of the ministry of education and each school authority. Then those teachers who were aged from 40 to 60, without sight difficulty and who were present in their working place during the data collection time were approached. Among the females, only those who gave informed written consent participated in the study. The group of teachers who participated in this study was first given a pre interventional test on July 01, 2018. Then the 99 participants were grouped into 10 groups in which nine groups contained 10 teachers except one.

Step 2: at this time, each group was called to the premises of Asmara College of health sciences for the provision of the training. This training was given for 3 days a total of 3 h for each group and the total time taken for the training was 1 month. The training was given by an expert midwife based on a teaching manual prepared by the investigator from several sources [36–39]. The teaching manual mainly focused on the definition of menopause, age at menopause, symptoms of menopause, physical activity and its benefit in reducing symptoms of menopause, nutrition, and what improvements should be employed when women reach menopause and post-menopause. The employed teaching methods were Lecture and group discussion. After the training, an immediate post-intervention test was done for each group, and the study participants sent home with handouts and brochures [36–39].

Step 3: After 3 months all study participants were called for a follow-up test and 98 came which made the response rate 98.9%.

### Instruments

The preliminary form of the questionnaire was adapted from an Iranian study [22] which was modified after reviewing relevant literature [20, 23, 40, 41]. Content and face validity was assured through evaluation of the questionnaire by various experts who are; 01 with Ph.D. in obstetrics and gynecology in nursing, 02 with MSc in midwifery, 01 professor in geophysics, 01 statistician with MSc in biostatics and epidemiology. In the Iranian study, the reliability of the questionnaire was determined by Cronbach’s Alpha which was 0.71 [22]. In this study, the internal consistency for knowledge (Cronbach’s alpha = 0.7) was acceptable [42]. Since the number of items used in the attitude scale was only nine, Cronbach’s alpha is not advisable to be reported instead of inter-item correlation [43]. Accordingly, the inter-item correlation ranged from 0.014 to 0.426, which are near the optimum recommended values by Briggs and Cheek ranging from 0.2 to 0.4 [43].

This questionnaire consisted of 39 items. The first eight items captured the socio-demographic characteristics of the study participants. Then 31 items were used to assess the knowledge (22 items) and attitude (9 items) regarding menopause. The items used to assess knowledge were selected based on the general knowledge of menopausal women. This included the definition of menopause, age at menopause, and those that affect it, menopausal symptoms, and health problems in the post-menopausal time. Moreover, the attitude items were selected based on women’s initiative to take care of themselves and their ability to lead unaffected life regardless of menopausal symptoms. There could be a potential overlap between the menopausal knowledge and attitude questions as found in this study.

Each correct response of the knowledge questionnaire was given a score of one and a wrong response a score of 0 that made an ideal maximum score of 22 and a minimum score of 0. Finally, the average score was used to compare the change of knowledge at three-time points (pre-intervention, immediate post-intervention and 3 months follow up). The attitude was assessed by nine questions put on Likert’s scale. The values of the 5-point scale ranged from one to five in which completely agree was scored one, agree = two neutral = three, disagree = four and completely disagree = five with the higher score showing a more positive attitude. The attitude assessing items was stated positively (four items) and negatively (five items). The positively stated questions were scored after the reversal of the scores (completely agree = five, agree = four neutral = three, disagree = two, and completely disagree = one). The mean score was used to compare the change in attitude at the three-time points.

Data collection in the three-time points was done by this questionnaire through a face-to-face interview.

### Statistical analysis

The data collected was coded, double entered, cleaned, and analyzed using Statistical Package for Social Sciences (SPSS, version 22.0). The normality of the entered data was checked by the Kolmogorov-Smirnov test and Fisher’s measures of skewness and kurtosis. To describe and summarize the study results, mean (SD) for continuous variables and frequency (percentages) for categorical variables were used. Mean score of knowledge and attitude towards menopause were computed at the pre-intervention immediate post and 3 months follow up. The change of scores at the three-time points on knowledge and attitude was investigated using repeated-measures ANOVA followed by pairwise Bonferroni post-hoc test. Factorial mixed ANOVA was used to determine the effectiveness of health education on knowledge and attitude of menopause across the categories of demographic variables. The results were presented using tables. A statistical significance was considered at *a p*-value of less than or equal to 0.05 except for the Box M *p*-value of less than or equal to 0.0001.

## Results

In this study, 110 middle age teachers were approached out of which 11 excluded due to failure to give consent and unwillingness to participate in this study. Finally, 99 middle age women participated in which one lost follow up that made the response rate 98.9%.

### Socio-demographic characteristics

The socio-demographic variables are shown in Table 1**.** The mean age of the study participants was 48.97(SD = 5.47) years and 77.8% of the respondents were married. Regarding the educational status of the respondents, 80(80.8%) of the respondents had a certificate and the remaining had a diploma and degree. Nearly all (98%) of the study subjects belonged to Tigrigna ethnic group. Most (82.8%) of the study participants were working in elementary schools while 13(13.1), 4(4%) worked in junior and secondary schools respectively. The majority (68.7%) of the study participants had a monthly income of $133.3–$166.6, 16.1% of the respondents had a monthly income of < $133.27 and only 15.2% had > $166.67monthly income.

**Table 1:** Sociodemographic characteristics of the study participants

Variable	Number	Percent
Age (Mean = 48.97, SD = 5.47)		
Educational Level		
Certificate	80	80.8
Diploma	16	16.2
Degree	3	3.0
Occupation		
Elementary school teacher	82	82.8
Junior school teacher	13	13.2
High school teacher	4	4.0
Marital status		
Married	77	77.8
Single	11	11.1
Divorced	8	8.1
Separated	3	3.0
Ethnicity		
Tigrigna	98	99.0
Saho	1	1.0
Religion		
Orthodox	81	81.8
Muslim	4	4.0
Catholic	8	8.2
Protestant	3	3.0
Other	3	3.0
Gross monthly salary		
< 66.67$	3	3.0
66.67$-133.27$	13	13.1
133.3$-166.6$	68	68.7
> 166.67$	15	15.2

### Knowledge about menopause parameters before and after the intervention

The result in Table 2 illustrates the proportion of women who correctly responded to the knowledge questions regarding the menopausal phenomenon. The percentage of women with knowledge on hereditary background affecting time of menopause were slightly greater than half (56.6%) at pre-intervention, however, over 90% of the study participants answered it correctly at immediate post-intervention (97.0%) and 3 months follow up (91.8%). How menstruation stops was only known by 26.3% of the study participants before the intervention but after intervention more than half (63.3% immediate post, 55.1% 3 months follow up) of the study participants knew how menstruation stops. Hot flush as a symptom of menopause was known by 65.7% of the study participants but almost all (94.9% immediate post-intervention and 96.9% 3 months follow up) of the study participants learned after the intervention. Those who knew about health problems related to menopause such as cardiovascular disease and osteoporosis were less than half (48.5% cardiovascular disease and 46.5% osteoporosis) at pre-intervention, however, they increased to 57 and 94% at immediate post-intervention and 56 and 90% respectively at 3 months follow up.

**Table 2:** Percent of knowledge parameters regarding menopause at the three-time points

Knowledge	Pre intervention (*n* = 99)	Immediate Post intervention (*n* = 99)	Three months follow up (*n* = 98)
	Correct	Correct	Correct
	n (%)	n (%)	n (%)
Menstruation stops suddenly^a^	38 (38.4)	44 (44.4)	38 (38.8)
Menopause occurs at the age of 48–55 years	75 (75.8)	85 (85.9)	89 (90.8)
Hereditary background affects the time of menopause occurrence	56 (56.6)	97 (97)	90 (91.8)
Menopause occurs due to increasing sexual hormones^a^	26 (26.3)	63 (63.6)	54 (55.1)
Menstruation disorder occurs before menopause	88 (88.9)	89 (89.9)	88 (88.8)
Most women experience hot flashes during the menopausal period	65 (65.7)	94 (94.9)	96 (96.9)
Menopause in women decreases genital infection^a^	31 (31.3)	57 (57.6)	59 (60.2)
Women in menopause increases weight and become obese	86 (86.9)	93 (93.9)	90 (91.8)
Menopausal symptoms are preventable and curable	40 (40.4)	64 (64.6)	42 (42.9)
Menopause decreases Cardiovascular diseases in women^a^	48 (48.5)	57 (57.6)	56 (57.1)
Menopause increases osteoporosis in women	46 (46.5)	94 (94.9)	90 (91.8)
Menopause causes dryness and skin shrivel in women	53 (53.5)	93 (93.9)	90 (91.8)
Sexualities change in menopausal women	65 (65.7)	82 (82.8)	79 (80.6)
Menopause increases extra hair on women’s face	10 (10.1)	73 (73.7)	67 (68.4)
Menopause causes vaginal dryness and painful sexual intercourse	46 (46.5)	89 (89.9)	85 (86.7)
Menopause causes urinary frequency and dysuria	48 (48.5)	91 (91.9)	78 (79.6)
Smoking and using alcohol increase osteomalacia in women	82 (82.8)	98 (99)	94 (95.9)
Regular physical activity is effective in preventing osteoporosis in menopausal women	81 (81.8)	98 (99)	93 (94.9)
Menopause affects the power of concentration and memory of women	78 (78.8)	93 (93.9)	91 (92.9)
The frequency and severity of hot flashes in menopausal women increase through time^a^	27 (27.3)	20 (20.2)	16 (16.3)
The level of stress and depressed feeling increases in menopausal women	62 (62.6)	90 (90.9)	87 (88.8)
Despite 1 year cessation of menstruation, pregnancy prevention is necessary^a^	69 (69.7)	50 (50.5)	49 (50)

^a^Negatively worded questions in which no was considered as correct answer

### Effect of health education on knowledge of menopause

Repeated measures ANOVA (Table 3) were used to assess the effect of educational intervention at pre-intervention, immediate post-intervention, and 3 months follow up. The mean scores of knowledge at pre-intervention, immediate post-intervention, and 3 months follow up were 12.3/22(SD = 3.06), 17.3/22(SD = 2.21), and 16.5/22(SD = 2.52) respectively. The difference of mean scores was found to be significant with time (Wilk’s value = 0.329, *p <* 0.0001). The change in knowledge scores showed a quadratic trend across the three-time points (*p* < 0.001).

**Table 3:** Effect of health education on knowledge scores regarding menopause

Time	Mean (SD)	Mauchly’s Sphericity (*p-*value)	Wilk’s Lambda	*p*-value	Trend (Partial Eta Squared)
Pre- intervention	12.3 (3.06)	0.800 (< 0.0001)	0.329	< 0.0001	Quadratic (0.589)
Immediate post intervention	17.3 (2.21)				
Three months later	16.5 (2.52)				

Pairwise comparisons (Table 4) of knowledge scores at three-time points (pre-intervention, immediate post-intervention, and 3 months follow up) were done using Bonferroni post-hoc test. The test showed that knowledge scores were significantly higher at immediate post-intervention than pre-intervention (*p* < 0.0001), at 3 months follow up than pre-intervention (*p < 0*.000) and immediate post-intervention than 3 months follow up (*p* = 0.004).

**Table 4:** Bonferroni post-hoc comparison of knowledge scores at the three-time points

Post-hoc Comparison	Mean Difference (95% CI)	*p*-value
Immediate post and pre-intervention	5.05 (4.17, 5.93)	< 0.0001
3 months follow up and pre-intervention	4.24 (3.35, 5.13)	< 0.0001
Immediate post and 3 months follow up	0.81 (0.21, 1.39)	0.004

### Effectiveness of educational intervention on knowledge across background characteristics

Factorial mixed ANOVA (Supplemental Table S1) was conducted to assess the impact of educational intervention on the scores of knowledge across three-time points (pre-intervention, immediate post-intervention and 3 months follow up) by age, educational level, and occupational level. There was no significant interaction between age and knowledge (Wilk’s Lambda = 0.949, *p* = 0.086), educational level, and knowledge within the three phases of intervention (Wilk’s Lambda = 0.997, *p* = 0.865). However, a significant interaction was observed between occupational level and knowledge within the three phases of intervention (Wilk’s Lambda = 0.842, *p* = 0.011). The educational intervention has shown a positive significant impact in increasing the scores of knowledge for those teachers working in elementary at immediate post-intervention than those working in junior and secondary school.

### The attitude of the study participants toward menopause

The result presented in Table 5 demonstrates the proportion of women who had a positive attitude towards the menopausal phenomenon. Menopause is the period of women’s loneliness was disagreed by 26.3% before the intervention, which then increased to 27.3, 34.3% at immediate post-intervention, and at 3 months follow up respectively. More than 80 % (84.4% pre-intervention, 86.9% immediate post-intervention, and 85.9% 3 months follow up) of study participants agreed with the statement that menopause was the period where problems of menstruation were eradicated in the three-time points. The ability of women to care for her-self using the information she has from different sources was agreed by 94.9% of the study participants before the intervention that increased to 97.0% at immediate post-intervention but then decreased to 92.9% at 3 months follow up. During the menopausal time, women’s attention towards her husband decreases was disagreed by 17.2% of the study participants at pre-intervention, which then increased to 18.2 and 25.5% at immediate post-intervention and at 3 months follow up respectively.

**Table 5:** Percentage distribution of attitude parameters towards menopause at the three-time points

Attitude	Pre intervention (n = 99)	Immediate Post intervention(n = 99)	Three months follow up (n = 98)
	Positive	Positive	Positive
	n (%)	n (%)	n (%)
Menopause is the period of woman’s loneliness^a^	26 (26.3%)	27 (27.3)	34 (34.3)
Menopause is the period of eradicating the Problem of menstruation	84 (84.8)	86 (86.9)	85 (85.9)
Menopause is the period where pregnancy is prevented	73 (73.7)	75 (75.8)	70 (71.4)
Women’s menopause decreases husbands sexuality^a^	42 (42.4)	44 (44.4)	43 (43.9)
women can care herself through the information she got from books, mass media, friends and others	94 (94.9)	96 (97.0)	91 (92.9)
During the menopausal time, interest and attention of women to her husband decreases^a^	17 (17.2)	18 (18.2)	25 (25.5)
Women become disable during menopause^a^	13 (13.1)	15 (15.2)	32 (32.7)
Women’s life in the menopausal period is more delightful than before menopause	41 (41.4)	42 (42.4)	33 (33.7)
Menopause decrease the grace of women’s appearance^a^	18 (18.2)	18 (18.2)	23 (23.5)

^a^Negative statements

### Effect of health education on the attitude of menopause

Repeated measures ANOVA (Table 6) were used to assess the effect of educational intervention on attitude at three-time points (pre-intervention, immediate post-intervention and 3 months follow up). The mean score of attitude before the intervention was 27.9/45(SD = 5.14), after the intervention the mean score became 28.3/45(SD = 5.25) and 28.32/45(SD = 5.12) at immediate post-intervention and 3 months follow up respectively. The difference among the mean scores was significant with time (Wilk’s value = 0.704, *p <* 0.0001).

**Table 6:** Effect of health education on attitude towards menopause

Time	Mean (SD)	Sphericity (*p*-value)	Wilk’s Lambda	*p*-value
Pre- intervention	27.9 (5.14)	0.774 (< 0.0001)	0.704	< 0.0001
Immediate post intervention	28.3 (5.25)			
Retention	28.3 (5.12)			

Pairwise (Table 7) comparisons of attitude scores at three-time points were also done using Bonferroni post-hoc test. The test showed that attitude scores were significantly higher at immediate post-intervention than pre-intervention (*p* = 0.001) and 3 months follow up than pre-intervention (*p* < 0.0001). However, there was no significant difference between mean scores of attitude at immediate post-intervention and 3 months later.

**Table 7:** Bonferroni post-hoc comparison of attitude scores at the three-time points

Post-hoc Comparison	Mean Difference (95% CI)	*p*-value
Immediate post and pre-intervention	0.43 (0.16, 0.69)	0.001
3 months follow up and pre-intervention	0.45 (0.25, 0.65)	< 0.0001
Immediate post and 3 months follow up	−0.02 (−0.29, 0.33)	1.000

### Effectiveness of educational intervention on attitude across background characteristics

Factorial mixed ANOVA (Supplemental Table S2) was conducted to assess the impact of the educational intervention on the scores of attitude across three-time points (pre-intervention, immediate post-intervention and 3 months follow up) by age, educational level, and occupational level. There was no significant interaction between age and attitude (Wilk’s Lambda = 0.997, *p* = 0.887), educational level, and attitude (Wilk’s Lambda = 0.997, *p* = 0.860), occupational level and attitude within the three phases of intervention (Wilk’s Lambda = 0.996, *p* = 0.808).

## Discussion

This study aimed at investigating the effect of health education on knowledge and attitude of menopause among middle-aged teachers. The study results demonstrated the positive impact of educational intervention on knowledge and attitude of menopause among female teachers.

### Knowledge about menopause parameters before and after the intervention

Acquiring Knowledge is an initial phase for the development of positive behavior and improvement of menopausal women’s quality of life [44–46]. When women’s awareness regarding menopause increases, it improves their attitude, health behavior, and health habits which eventually lead to an improvement in their quality of life [47]. In this study, the mean knowledge score regarding menopause significantly improved after the educational intervention. Similarly in Egyptian studies, the total knowledge score increased significantly after the health educational program [20, 23]. Contradicting to both results, an Iranian study reported that there was no significant change in the mean score of knowledge from pre-intervention (10.52) to post-intervention (15.14) [32]. This difference in findings might be due to the different educational materials utilized and the presence of study participants with a lower educational level in the comparative study.

In this study, those women who were aware of the appearance of unwanted hair on the face and the level of natural hormonal levels during menopause were below 30% before the training. This could be attributed to the lack of educational information provided to women in Eritrea regarding menopause. Menopause has not been seriously handled even by the responsible sections in the Ministry of Health, which led to the vicious cycle of women repeatedly seeking medical help to relieve symptoms. In addition to this, studies showed that the information they receive is not scientific as most of it comes from friends and relatives [28]. An encouraging finding in this study was that most of the study participants were aware of menstrual irregularity as a symptom of menopause, weight gain during menopause, the prevention of osteoporosis during menopause through physical activity, smoking and alcohol drinking as a risk factor of osteoporosis before the intervention. This was similar to various studies [20, 22, 23, 28].

After the intervention, an increase in the knowledge of the study participants was seen on all items of the knowledge questions. However, still, the highest improvement was seen on the knowledge of the effect of hereditary background on the time of menopause occurrence, the effect of menopause on osteoporosis, increase extra hair on the face, skin dryness and shrivel, urinary frequency, dysuria, and hot flushes as a symptom of menopause. The increase in the knowledge about menopause suggests the usefulness of the given training. Similarly, in a study done in Egypt, the highest level of improvement was found in the knowledge about the definition of menopause, weight gain, osteoporosis, urinary incontinence, and dyspaurina [20].

In this study age had no significant association with change in knowledge; this was similar to what was reported in a study conducted in Egypt [20]. The educational level too did not have a significant association with the change in knowledge of the study participants. However Egyptian studies found out that educational level had a significant effect in the improvement of knowledge [20, 23]. The possible reason for this difference could be the homogeneity (all teachers) of the study participants in this study, unlike the comparable studies whose participants had various educational backgrounds. The improvement of knowledge was significantly higher in those working in elementary school than those working in middle and secondary schools in the present study. The above Egyptian studies also found a significant effect of an occupational level to knowledge improvement across time [20, 23].

### Attitude toward menopause before and after the intervention

Attitude towards menopause was one of the factors that affect women’s quality of life during the period [26]. As women attain a more favorable attitude towards menopause their quality of life also improves [1, 48–50]. Moreover, women with a positive attitude are reported to have lower severity of menopausal symptoms [30]. Further to this, attitude is an effective organizing principle in acting and can start a health behavior due to the effect it has on the person [51]. In this study, the intervention brought a significant increase in the mean scores of attitude across time. Among other factors, women’s attitude towards menopause is affected by their involvement in educational programs which can help them to be self-sufficient and take care of themselves [26]. This was in agreement with studies done in Egypt which showed a significant increase in positive attitude after the educational intervention [20, 23]. Whereas in a study done in Iran, there was no significant change in attitude after the educational intervention. In this study knowledge of the participants was not affected by the education given so the unchanged attitude of the participants towards menopause is highly expected [32].

The present study showed that there was no significant relationship between improvement of attitude and participant’s age across time. This result is similar to another study done in Iran by Taherpour et al. (2015) in which age did not affect the improvement of attitude and concluded saying that women with good knowledge tend to have a positive attitude toward menopause regardless of their age. This study has found no change in attitude after educational intervention across the categories of educational and occupational levels. This finding is similar to Egyptian studies conducted by Elnaggar et al. (2013) and Orabi (2017) in which they discovered a lack of change as attitude needs more time to adjust than knowledge needs and so stressed the need for timely and repetitive education to achieve the goal [20, 23].

Before the intervention, the majority (84.8, 73.7%) of study participants believed menopause to be a comfortable time to prevent problems of menstruation and pregnancy respectively. This was mentioned by 67% of women in Iranian study [22]. In the same study, 81.5% had a positive attitude towards menopause and 70% agreed with the fact that women’s lives in the menopausal period are more delightful than before. In this study, almost all (94.9%) agreed with the fact that women can care for themselves using the information they have from others. Even though this is the case, women in Eritrea did not have any information about menopause from mass media or other menopausal centers. The results seem to be reassuring in the sense that the majority of women seem to have a positive attitude towards menopause and are ready to take the necessary measures to improve their health status and experience.

### Study limitation

The main limitation of this study was the lack of a control group and some items of the knowledge questionnaire were not clear as they indicated all women experience the same symptoms. Moreover, as this study was preliminary in Eritrea it was not as culture-sensitive as it should be. So we recommend the performance of a more culturally sensitive study using this study as a baseline. The Attitude questions are also difficult to generalize to other settings so a more generalizable questionnaire is recommended for future study. Though this study was conducted among 99 female teachers, it could give us some image on the status of menopausal women in Eritrea. If the knowledge and attitude of this educated group of women were low and improved with health education, this intervention would benefit the majority of uneducated Eritrean women. The same study could be made with a control group and a standardized questionnaire.

## Conclusion

Women’s knowledge about the common symptoms of menopause, their health problems during menopause, and prevention mechanisms were very low before the intervention. A significant increase was noted after the intervention. The study participants’ level of positive attitude had significantly improved after the educational intervention. At the immediate post-intervention phase, the knowledge of teachers working in elementary schools was more impacted than those teachers working in middle or secondary. Interventions such as this could be easily employed to raise awareness of women, instill a favorable attitude, and hence promote good quality of life during menopause.

## Supplementary information

**Additional file 1 : Supplemental Table S1**. table of effectiveness of educational intervention on knowledge across background characteristics. **Supplemental Table S2**. table of effectiveness of educational intervention on attitude across background characteristics.

## Data Availability

The complete dataset used and/or analyzed during the current study are available from the corresponding author and can be accessed upon reasonable request.

## Abbreviations

KA: Knowledge and attitude
HRT: Hormonal replacement therapy
