# Supplementary material for: Effect of health education on knowledge and attitude of menopause among middle-age teachers
Source: BMC Womens Health. 2020 Oct 12;20:232. doi: 10.1186/s12905-020-01095-2 (PMC7552533; doi:10.1186/s12905-020-01095-2)
Supplement: Supplementary file 1 — Additional file 1 : Supplemental Table S1. table of effectiveness of educational intervention on knowledge across background characteristics. Supplemental Table S2. table of effectiveness of educational intervention on attitude across background characteristics. [file 12905_2020_1095_MOESM1_ESM.docx]

**Supplemental Material**

**Supplementary Table S1: Effectiveness of educational intervention on knowledge of study participants by background characteristics.**

| Characteristics | | Pre-intervention | Immediate Post | 3-Months | Box M  *p*-value | Wilk's Lambda | *p*-value |
| --- | --- | --- | --- | --- | --- | --- | --- |
|  |  |  |  | Follow up |  |  |  |
|  |  | M (SD) | M (SD) | M (SD) |  |  |  |
| Age |  |  |  |  |  |  |  |
|  | 40 to 50 | 11.97(3.24) | 17.32(2.01) | 16.79(2.56) | 0.111 | 0.949 | 0.086 |
|  | 51 to 60 | 12.97(2.59) | 17.29(2.54) | 16.14(2.44) |  |  |  |
| Occupational Level | |  |  |  |  |  |  |
|  | Elementary | 12.19(3.11) | 17.35(2.27) | 16.44(2.45) | 0.951 | 0.989 | 0.011 |
|  | Junior or Secondary | 12.71(2.89) | 17.24(1.99) | 16.94(2.88) |  |  |  |
| Educational Level | |  |  |  |  |  |  |
|  | Certificate | 12.19(3.13) | 17.32(2.09) | 16.46(2.39) | 0.556 | 0.997 | 0.865 |
|  | Diploma or Degree | 12.68(2.81) | 17.42(2.69) | 16.84(3.06) |  |  |  |

**Supplemental Table S2: Effectiveness of educational intervention on attitude across background characteristics.**

| Characteristics | | Pre-intervention | Immediate Post | 3-Months | Box M  *p*-value | Wilk's Lambda | *p*-value |
| --- | --- | --- | --- | --- | --- | --- | --- |
|  |  |  |  | Follow up |  |  |  |
|  |  | M (SD) | M (SD) | M (SD) |  |  |  |
| Age |  |  |  |  |  |  |  |
|  | 40 to 50 | 27.94(4.64) | 28.34(4.89) | 28.66(5.41) | 0.012 | 0.997 | 0.887 |
|  | 51 to 60 | 27.97(5.89) | 28.46(5.80) | 29.03(5.06) |  |  |  |
| Occupational Level | |  |  |  |  |  |  |
|  | Elementary | 28.01(5.30) | 28.42(5.39) | 28.79(5.63) | 0.024 | 0.996 | 0.808 |
|  | Junior or Secondary | 27.18(4.35) | 27.71(4.62) | 28.59(2.89) |  |  |  |
| Educational Level | |  |  |  |  |  |  |
|  | Certificate | 27.85(5.21) | 28.28(5.30) | 28.59(5.57) | 0.048 | 0.997 | 0.860 |
|  | Diploma or Degree | 27.95(4.95) | 28.37(5.19) | 29.42(3.69) |  |  |  |
